# Affective and reflective attitudes toward vegetarian food consumption: the effect of goal priming

**DOI:** 10.3389/fnut.2025.1653935

**Published:** 2025-11-06

**Authors:** Fabian Daiss, Petra Jansen

**Affiliations:** Institute for Sports Science, Faculty of Human Sciences, University of Regensburg, Regensburg, Germany

**Keywords:** goal priming, implicit attitudes, explicit attitudes, vegetarian nutrition, sustainable behavior, dual-process models, evaluative readiness

## Abstract

**Objectives:**

The study’s primary goal was to investigate the effect of goal priming on implicit and explicit attitudes toward vegetarian food consumption and food choice behavior within the context of dual-process models that describe sustainable behavior.

**Methods:**

A total of 128 participants were randomly assigned to either a goal priming intervention group or a control group. After reading a short priming text, all participants completed an explicit rating task, an Implicit Association Test (IAT), and a simulated online supermarket task to assess actual food-related choices.

**Results:**

Participants in the intervention group exhibited significantly more positive implicit attitudes toward vegetarian food compared to those in the control group. Explicit attitudes toward vegetarian food were also significantly more positive in the intervention group, although to a smaller extent. No significant group differences were found in explicit attitudes toward meat-based nutrition or in food choice behavior. However, mediation analysis revealed a significant indirect effect of goal priming on behavior via implicit attitudes. Exploratory analyses showed consistent gender differences across all outcome variables, which attenuated the priming effects when included as a covariate.

**Conclusion:**

Although the intervention did not result in direct behavioral change, the findings support the potential of goal priming to influence automatic affective and reflective processes that may precede the development of sustainable behavior.

## 1 Introduction

The production and consumption of food strongly influences worldwide greenhouse emissions and can be reduced by abstaining from meat consumption (1, 2). To relieve the current environmental challenges, sustainable behavior must be enforced. Recent large-scale analyses demonstrate that sustainable food choices require considering full life-cycle environmental impacts of both plant- and animal-based products (3). Cross-cultural research further indicates that not only sustainability knowledge but also psychological traits such as impulsivity modulate the frequency of animal- and plant-based food consumption, with higher knowledge being associated with greater plant-based intake and reduced animal consumption (4). Complementing these findings, an interdisciplinary review emphasizes that sustainable food consumption should be conceptualized as a goal-directed challenge involving behavioral, affective, and cognitive dimensions (5), while recent consumer segmentation studies highlight that environmental attitudes and knowledge are directly linked to real-world preferences for circular and sustainable food systems across Europe (6). Further literature highlights the importance of automatic or non-conscious cognitive processes, in addition to reflective and conscious processes, and therefore emphasizes the necessity of dual-process models to describe behavior, such as the Affective-Reflective Theory (7). These automatic (affective, implicit) or conscious (reflective, explicit) processes are based on either affective or reflective attitudes (7). Following this, behavior can be altered by changing attitudes (8, 9), and as a result, sustainable behavior is empowered through improving attitudes toward vegetarian nutrition. One promising way to influence affective and reflective attitudes and behavior is through goal priming (10). Recent reviews show that goal priming can activate valued health goals and guide choices in everyday contexts (11, 12). Experimental work demonstrates that goal priming shifts attention toward goal congruent foods and increases healthier choices in realistic shopping tasks (13). A systematic review and meta-analysis indicates small but reliable effects of weight control and health primes on eating outcomes (14).

### 1.1 Theoretical frameworks of sustainable behavior

Following (52), sustainable behavior can be conceptualized as a blend of self-interest and prosocial concern. While earlier theoretical frameworks often prioritized one of these dimensions, such as prosocially oriented models [e.g., the norm-activation model, Schwartz (53)] or self-interest-based models [e.g., the theory of planned behavior, Ajzen (54)], Klöckner and Blöbaum’s (55) Comprehensive Action Determination Model (CADM) offers a more integrative perspective. It combines normative influences [e.g., social norms, personal values) with rational decision-making components (e.g., perceived behavioral control) into a unified structure.

However, these models primarily describe behavior as the outcome of deliberate and reflective processes, shaped by consciously held values (e.g., egoistic, altruistic, biospheric, or hedonistic) and intentions. They largely neglect the role of automatic, situationally triggered processes, which can influence behavior outside of conscious awareness (15, 56). To address this limitation, dual-process models have gained relevance in sustainability research.

One such model is the Affective-Reflective Theory of Physical Inactivity and Exercise [ART; (7)], which builds upon dual-process theories of cognition (57, 58). ART posits that behavior is the result of two interacting systems: a type-1 process, characterized by fast, automatic, and affect-driven responses to stimuli, implicit attitudes, and a type-2 process, involving slower, deliberate, and reflective evaluations, as well as explicit attitudes. The type-1 process generates an automatic affective valuation, which can trigger an action impulse, whereas the type-2 process leads to a reflective evaluation, forming the basis for an action plan (7).

When a discrepancy arises between the affective and reflective responses, and self-control resources are depleted, behavior is more likely to be dominated by the affective, type-1 process. One method to influence these processes is through goal priming.

### 1.2 Goal priming

As a result of the intention-behavior gap, long-term pro-environmental goals tend to be overshadowed and overruled by salient short-term goals (10). However, goals can be reactivated, both consciously and non-consciously (16), producing behavior, perceptions, and judgments that align with the primed goal. Experimentally activated goals lead to temporary changes in the automatic evaluation of objects instrumental to goal attainment (17, 18) and, therefore, the implicit attitudes toward them. The malleability of automatic evaluation following goal activation is called evaluative readiness. Reading task instruction is one possible way to (re-) activate goals (10). Evaluative readiness arises from the increased accessibility of positive memories and knowledge structures linked to goal-relevant stimuli. When a goal is activated, memories and knowledge with positive associations to objects that facilitate the attainment of that goal become more accessible, while inhibiting corresponding negative memories. It has been demonstrated that mental accessibility of environmentally related constructs is crucial for sustainable and pro-environmental behavior (15, 19, 20). Thus, as implicit processes serve as the basis for explicit evaluations (7, 29), goal priming is a promising way to influence both implicit and explicit attitudes.

### 1.3 Explicit and implicit attitudes in sustainable behavior

Attitudes can be understood as our conscious or subconscious assessments of objects, behaviors or situations. Dual-process models emphasize that human behavior is guided by both controlled, reflective processes and automatic, affective processes (59, 60). Following ART, these two systems are typically aligned with type-2 and type-1 processes, respectively.

Explicit attitudes refer to evaluations that individuals can deliberately access and report. They are typically assessed using self-report instruments, such as semantic differentials or Likert-type scales, where individuals consciously evaluate, for example, vegetarian versus meat-based meals. These assessments capture the reflective, propositional system of attitude processing (21).

In contrast, implicit attitudes reflect automatic and often unconscious evaluations, which may not be accessible through introspection. These attitudes can be assessed using indirect measures such as the Implicit Association Test [IAT; (22)].

The distinction between explicit and implicit attitudes is especially relevant in the context of sustainable behavior, as individuals may hold pro-environmental beliefs at a reflective level while simultaneously harboring affective preferences for unsustainable options. Such discrepancies can explain attitude-behavior gaps, where reported intentions do not align with actual choices (23). Recent work in food cognition shows that both explicit and implicit evaluations of plant- and animal-based foods are informative and meaningfully connected to eating behavior. For instance, explicit ratings tend to track one’s habitual diet while implicit affective evaluations often favor vegetarian foods across groups (24). Extending this, explicit and implicit pro-vegetarian attitudes link to sustainable consumption tendencies (25) and show that combining implicit and explicit measures provides nuanced insights into acceptance of novel sustainable proteins, such as insect-based foods (26).

### 1.4 The goal of the study

This study aims to examine the effects of goal priming focused on vegetarian nutrition, seeking a new approach to support sustainable consumption behavior. According to the United Nations (27), plant-based nutrition can significantly contribute to combating climate change. We investigate how goal priming activating a pattern of positive automatic associations and long-term goals with vegetarian nutrition, influences the explicit and implicit attitudes toward vegetarian and meat-based nutrition and consequential behavior using an explicit rating task, an implicit association test [IAT; (22)] and an online supermarket task (28).

Tate et al. (10) demonstrated that implicit attitudes can be modified through brief goal priming, facilitated by evaluative readiness. Following ART (7), implicit attitudes form the basis of automatic associations, which is the primary component of the unconscious Type-1 process. Goal priming can elicit evaluative readiness due to the heightened accessibility of positive memories and knowledge structures associated with the goal, while inhibiting negative memories with goal-relevant stimuli, resulting in more positive automatic associations. Therefore, we formulate the following hypothesis:

*H1*: Implicit attitudes toward vegetarian nutrition are more positive for the intervention group than for the control group.

Reflective evaluations are based on automatic associations (7, 29) in the form of a default-interventionist model, in which the affective valuation is the default response upon which the slower, controlled response is based. Furthermore, goal priming also points out positive information about vegetarian nutrition to influence propositions, like one’s needs and values, pros and cons of behavioral change, beliefs, morals, and social expectations to reach long-term goals, which are part of the type-2 process with explicit attitudes (7). Furthermore, the goal priming of the intervention group contains several adverse facts about the production and consumption of meat-based foods, also influencing propositions about meat-based nutrition. Therefore, we hypothesize:

*H2*: Explicit attitudes toward vegetarian nutrition are more positive for the intervention group than for the control group. Explicit attitudes toward meat-based nutrition are more negative for the intervention group than for the control group.

Tate et al. (10) demonstrated that activating environmental goals led to a shift in consumers’ behavior toward the greener option by altering the automatic valuation of goal-relevant stimuli. Therefore, the following hypothesis was formulated:

*H3*: There is a more positive vegetarian nutritional behavior in the intervention group than in the control group, measured by an online supermarket task. Implicit and explicit attitudes mediate the effect of goal priming on choice behavior.

## 2 Method

We aim to investigate the impact of goal priming on the explicit and implicit attitudes toward vegetarian and meat-based nutrition. A between-subjects design was applied, comparing vegetarian nutrition goal priming with comparison identity fraud goal priming. The participants completed the intervention (group-dependent goal priming), followed by the explicit evaluation task, the IAT, the supermarket task, and the demographic questionnaire.

The study adhered to the principles of the Helsinki Declaration regarding ethical guidelines and was approved by the University of Regensburg’s Ethical Board (reference number: 20-1978_4–101).

### 2.1 Participants

For an appropriate sample size, a power analysis, calculated using G*power (30), for t-tests comparing the difference between two independent means (two groups) with a medium effect size of d = 0.5 [typically observed in priming; research (31)], an alpha-level of 0.05 and a power of 1-ß = 0.80 resulted in *N* = 128 to detect significant differences between the condition vegetarian nutrition goal priming or comparison identity fraud goal priming in explicit and implicit attitudes toward images of vegetarian and meat-based nutrition and consumer behavior (Hypotheses 1, 2 and 3a).

With an effect size of *f*^2^ = 0.15 (31), an alpha level of 0.05, and a power of 1-*β* = 0.80, a power analysis for the mediation analysis to determine whether changes in implicit or explicit attitudes mediate the effect of goal priming on choice behavior resulted in *N* = 55 (Hypothesis 3b).

All participants were randomly assigned to one of two groups (experimental goal priming or comparison group), resulting in equally sized samples for both groups.

Participants were eligible if they were enrolled in the Applied Movement Science program at the University of Regensburg, were 18 years of age or older, and reported no reading difficulties. Individuals not meeting these criteria or indicating reading difficulties were excluded. All participants provided informed consent, were recruited via social media or the institute’s newsletter and gained study credits for their participation. No participants were excluded for attention or comprehension failures; a brief free-text paraphrase of one action tip from the priming text, administered at the end of the demographic questionnaire, documented comprehension for all respondents. As a result, no exclusions affected group balance or statistical power.

### 2.2 Material

In this study, a goal priming intervention, an explicit affective evaluation, an implicit association test, a supermarket task, and a demographic questionnaire were applied.

### 2.3 Goal priming task

According to Tate et al. (10), goals are explicitly primed using text vignettes related to a reading comprehension task. The text features a ‘problem’, eight facts about the causes and consequences of the problem, and five tips to avoid this problem inspired by recommendations of the German Nutrition Society (DGE). The text contains approximately 350 words. The environmental goal prime promoting vegetarian nutrition describes the causes and environmental impact of growing meat production. The differences between vegetarian and meat-based food production are mentioned several times to ensure that vegetarian nutrition is viewed as instrumental in achieving the environmental goal. The neutral goal prime focuses on discussing the causes, prevalence, and financial consequences of identity fraud. It has the same text structure, maintaining consistency across conditions. The goal to safeguard one’s financial resources is considered relatively universal and potentially of equal or greater significance compared to the environmental goal. Both primes introduce their respective topics using identical wording.

To verify that the material had been processed, a brief comprehension check was administered at the end of the demographic questionnaire. This check required a free-text response in which participants paraphrased one of the five tips in their own words. All participants provided a substantive paraphrase, indicating successful reading and understanding of the prime. Placing the check at the end of the questionnaire limited demand characteristics during the immediately following tasks while still documenting comprehension of the priming material.

### 2.4 Explicit evaluation task

For the explicit rating task, five pictures of meat-containing food and five pictures of vegetarian food were selected from Blechert et al.’s (32) database, matched in terms of familiarity, arousal, and valence. Stimulus characteristics are provided in Supplementary Table 1, and the machine-readable dataset together with the original images is available on OSF.^1^ The label “vegetarian” is used in the lacto-ovo sense, that is, foods without meat or fish. In the stimulus set, two of the five vegetarian images displayed dairy in the form of cheese, and three contained no animal derived ingredients; none depicted eggs. This distinction is highlighted because environmental impacts and nutritional profiles of dairy based items can differ from strictly plant-based alternatives, and such differences may shape consumer evaluations (33, 34). The explicit evaluation rating task consisted of the following question: “What is your attitude to the following picture?” (1 = “negative,” 7 = “positive”). Participants had 5 sec to respond to provoke a spontaneous reaction. Explicit attitude indices for vegetarian and meat-based foods were defined as mean ratings across the five images per category. Item level ratings were not retained in the final analysis file. Therefore, internal consistency estimates for these composites cannot be computed retrospectively. The decision to aggregate across multiple exemplars was made *a priori* to reduce item specific variance.

### 2.5 Implicit association test

The standard Implicit Association Test (IAT) was chosen to assess implicit attitudes (22). The IAT used in this study was adapted from the version of Winkelmair and Jansen (61). It comprises four categories: two target categories and two attribute categories, along with various stimuli, including target images and attribute words. As target categories, “vegetarian” and “meat” were used, and as attribute categories, “positive” and “negative” were employed. Target categories denoted food types and were presented as “vegetarian” and “meat.” In the present context these labels referred to vegetarian food and meat-based food, respectively, rather than to dietary identities. As target images and attribute words, we used the same 10 pictures of vegetarian or meat-based foods as in the explicit affective evaluation, along with five positive and five negative words from the Berlin Affective Word List (35). Consistent with the explicit evaluation task, the label “vegetarian” in the IAT refers to the same lacto-ovo vegetarian set of five images, which included two items with cheese and three strictly plant-based items. Participants were instructed to sort images or words appearing in the center of the screen by pressing the left (“D”) or right (“K”) key, depending on the assigned categories.

**Figure 1:**
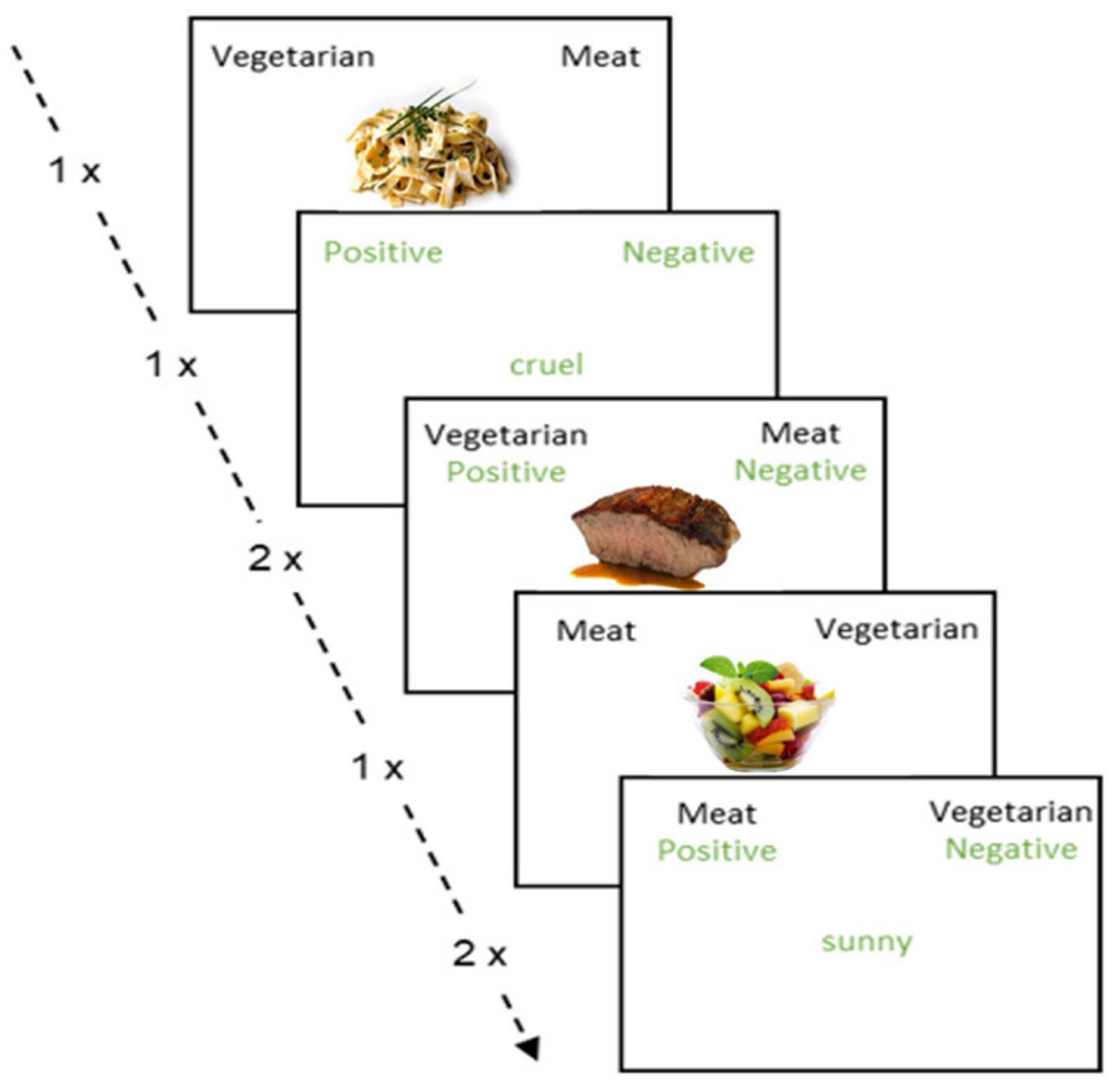
Experimental setting of the implicit association test.

The task followed the standard seven block IAT structure with a total of 180 trials. Blocks 1 and 5 presented only target images (20 trials each). Block 2 presented only attribute words (20 trials). Blocks 3 and 6 were combined blocks with 20 trials each, and Blocks 4 and 7 were combined blocks with 40 trials each. In combined blocks, a target label in black font and an attribute label in green font appeared on each side of the screen. On each trial, a single stimulus was shown in the center and was categorized by pressing the left key “D” or the right key “K,” according to the labels shown at the top (see Figure 1). Target images and attribute words alternated within combined blocks, with target images on odd trials and attribute words on even trials. Category sides were randomized across participants and were reversed at the start of Block 5 for the remainder of the task. Incorrect responses produced a red cross until the correct key was pressed, and the latency from the initial press to the subsequent correct press was recorded. Trials from Blocks 1, 2, and 5 were not used for scoring. Trials with response times greater than 10,000 ms were excluded. Participants with more than 10 percent of trials faster than 300 ms were excluded. Error trials were retained using a built in penalty (36), defined as the sum of the latency for the incorrect response and the latency for the immediately following correct response. Implicit attitudes were indexed with the D-score (36). Separate D-scores were computed for short combined blocks and long combined blocks by subtracting the mean latency of compatible trials from the mean latency of incompatible trials and dividing by the pooled standard deviation of latencies for the respective block lengths. These two values were then averaged to produce the final D-score. The runnable task script and parameter files, as well as example stimuli, are available at the project repository on OSF (see text footnote 1, respectively).

Split half reliability was estimated from short and long D-components using the Spearman Brown correction. The resulting estimate was *ρ* = 0.81, 95% CI [0.74, 0.87], *N* = 128.

### 2.6 Supermarket task

The online supermarket task (28) is a behavioral measure that investigates food product choices in an online supermarket setting. The task comprises 170 products assigned to eight different product categories (bread, rice, pasta, and other grains; spreads and cereals; eggs and dairy; ready meals; meat, poultry, fish, and seafood; fruits and vegetables; sweet and salty snacks; oils, sauces, nuts, and legumes) with 16–26 products per category. Participants received the instruction as follows: “You want to buy food for yourself in the online supermarket for the next few days. Please choose 20 products”.

The photographic stimuli used in the IAT and the explicit evaluation task were not presented again in the supermarket. The supermarket measured choices at the category and product level in order to avoid recognition or habituation to specific pictures and to reduce demand characteristics from stimulus repetition. Some ingredients conceptually overlapped with those depicted in the rating and IAT sets, yet the exact images and item presentations differed.

The number of meat-based products chosen in the online supermarket task was used as an indicator for sustainable behavior and vegetarian nutrition behavior.

### 2.7 Demographic questionnaire

Participants answered questions concerning sex, age, education stage, importance of nutrition, importance of sustainable nutrition, eating habits (vegan, vegetarian, omnivorous), current income, and one question about the goal priming text as part of the text comprehension task was asked. Eating habits was assessed with three response options, but for analysis, vegan responses were collapsed into the vegetarian category because the stimuli in the attitude tasks followed a lacto-ovo definition and included two items with dairy. Primary analyses therefore used a two-level indicator (omnivore vs. vegetarian/vegan). Given the unequal distribution of eating-habit groups, eating habit was summarized descriptively and not used as a primary grouping factor in the main tests of the priming effect.

### 2.8 Procedure

The experiment lasted 30 min and was conducted using the programs OpenSesame (62) and JATOS (37). The participants began with goal priming for vegetarian nutrition or control identity fraud, followed by explicit affective evaluation and the implicit association test. Following these tests, the supermarket task and the demographic questionnaire were conducted. Each participant was tested individually in a quiet, undisturbed room to ensure standardized conditions and minimize external influences.

### 2.9 Statistical analysis

Hypotheses and the analytic plan were specified before data collection in the preregistration at OSF: https://osf.io/hmkcn/. Deviations from the preregistration are marked as exploratory. Descriptively, demographic variables, e.g., age and gender distributions, are reported. Furthermore, variables of interest to our present study, such as the numbers of vegetarians and omnivores, and the general importance of their nutrition, are reported. All participants supplied a valid free-text paraphrase on the comprehension check of the intervention. Therefore, no exclusions were made on this basis. To test, if there are significant differences in implicit on the one side and explicit attitudes on the other side toward images of vegetarian and meat-based nutrition, between-participant comparisons were analyzed using independent t-tests, each individual for the dependent variables implicit and explicit attitude (H1 and H2) and the independent variable group (between, vegetarian nutrition goal priming vs. comparison identity fraud goal priming).

To test H3, which posits that changes in explicit and implicit attitudes mediate the effect of goal priming on choice behavior, we conducted an independent t-test with the dependent variable of behavior choice and the independent variable of group to determine if there is a significant difference in food choice between the intervention and control groups. Following, we conducted a mediation analysis using the PROCESS macro by Hayes (38), which uses ordinary least squares regression, to test if the effect of goal priming (independent variable; vegetarian nutrition goal priming/comparison identity fraud goal priming) on choice behavior (dependent variable) gets mediated by explicit and/or implicit attitudes.

Given the achieved sample size of *N* = 128 and *α* = 0.05, a *post hoc* sensitivity estimate indicates approximately 80% power to detect effects of d ≈ 0.50 in between group comparisons.

Brief exploratory checks addressed an observed gender imbalance and distributional features. A univariate general linear model with group and gender yielded group effects consistent in direction with the preregistered analyses across outcomes: IAT D-score coefficient = 1.26, 95% CI [−0.22, 2.734], *p* = 0.094; explicit attitudes toward meat coefficient = −0.16, 95% CI [−0.75, 0.43], *p* = 0.588; explicit attitudes toward vegetarian foods coefficient = 0.19, 95% CI [−0.18, 0.55], *p* = 0.313. For the count outcome number of meat items, Poisson models indicated overdispersion (Pearson *χ*^2^ per degree of freedom ≈ 1.71), therefore a negative binomial model was used, yielding a rate ratio for intervention versus control of 0.93, 95% CI [0.59, 1.48], *p* = 0.775. A Mann Whitney test converged on the same pattern (*U* = 2,375, *p* = 0.108). These checks did not change the direction of the preregistered results and are reported as exploratory.

## 3 Results

### 3.1 Demographic data

There is a statistically significant difference between men and women regarding age [*t* (126) = 2.87, *p* = 0.005], with women being, on average, 2.04 years older than men (95% CI [0.63, 3.45]). There is no statistical difference between men and women regarding the importance of nutrition [*t* (126) = 1.71, *p* = 0.089], the importance of sustainable nutrition [*t* (123.93) = −1.67, *p* = 0.098], and eating habits [*χ*^2^ (2) = 3.21, *p* = 0.201]. The exact statistical values are reported in Table 1.

**Table 1:** Means (SD) of age, importance of nutrition (Imp), importance of sustainable nutrition (Imp sus), nutritional behavior (NB: amount of chosen meat-based products) implicit attitudes and explicit attitudes (Meat: explicit attitudes toward meat-based food and Veg: explicit attitudes toward vegetarian food) and relative frequency of eating habits (O: omnivore, V: vegetarian).

Gender	Age	Imp	Imp sus	Eating habits	Implicit attitudes	Explicit attitudes	NB
Men (*N* = 66)	23.58* (5.06)	5.65 (1.08)	4.44 (1.30)	O: 77.3% V: 22.7%	0.42* (4.47)	Meat 4.18* (1.64) Veg 5.25* (1.02)	2.50* (1.97)
Women (*N* = 62)	21.53* (2.50)	5.32 (1.08)	4.79 (1.07)	O: 62.9% V: 37.1%	4.12* (3.59)	Meat 2.94* (1.55) Veg 5.85* (0.98)	0.94* (1.30)

Significant group differences (*p* < 0.05) are indicated with an asterisk (*).

Furthermore, there is no statistical difference between participants of the intervention and the control group regarding age [*t* (126) = −0.28, *p* = 0.783], importance of nutrition [*t* (126) = −0.80, *p* = 0.936], importance of sustainable nutrition [*t* (126) = 0.73, *p* = 0.465], and eating habits [*χ*^2^ (2) = 0.41, *p* = 0.816]. Conversely, a statistical difference exists between the intervention and control groups regarding gender [*χ*^2^ (1) = 8.01, *p* = 0.005].

Dietary composition of the sample was as follows: 90 participants were omnivores (70.3%) and 38 were vegetarian or vegan (29.7%) in the combined sample (*N* = 128). By gender, men included 51 omnivores and 15 vegetarian/vegan respondents (*N* = 66), while women included 39 omnivores and 23 vegetarian/vegan respondents (*N* = 62). Owing to the unequal sizes of the omnivore and vegetarian or vegan groups, no inferential comparisons were conducted between these dietary groups; primary analyses focused on the effect of experimental condition in the full sample.

### 3.2 Implicit attitudes toward vegetarian nutrition

There were no outliers in the data. There were 64 participants in each group (*N* = 128). Implicit attitudes toward vegetarian food were higher in the goal priming intervention group (*M* = 3.27, SD = 4.18) than in the control group (*M* = 1.15, SD = 4.51, see Table 2). There was a statistically significant difference between implicit attitudes toward vegetarian food of the goal priming group and the control group, with higher implicit attitudes scores [95%-CI (−3.64, −0.60)] for the goal priming group, *t* (126) = −2.76, *p* = 0.003, *d* = −0.49.

**Table 2:** Descriptives of implicit attitudes toward vegetarian nutrition (Imp_Veg), explicit attitudes toward vegetarian (Exp_Veg), meat-based nutrition (Exp_Meat) and nutritional behavior (NB, amount of chosen meat products in supermarket tasks).

Variable	Group	*N*	Mean	SD
Imp_Veg	Intervention	64	3.27*	4.18
	Control	64	1.15*	4.51
Exp_Veg	Intervention	64	5.70*	1.04
	Control	64	5.38*	1.03
Exp_Meat	Intervention	64	3.35	1.72
	Control	64	3.81	1.68
NB	Intervention	64	1.55	1.89
	Control	64	1.94	1.81

Significant group differences (*p* < 0.05) are indicated with an asterisk (*).

### 3.3 Explicit attitudes toward vegetarian and meat-based nutrition

The evaluation of the data on explicit attitudes toward vegetarian nutrition showed one outlier remaining in the dataset. There were 64 participants in each group (*N* = 128). Explicit attitudes toward vegetarian food were higher in the goal priming intervention group (*M* = 5.70, SD = 1.04) than in the control group (*M* = 5.38, SD = 1.03, see Table 2). There was a statistically significant difference between explicit attitudes toward vegetarian food of the goal priming group and the control group, with higher explicit attitudes scores [95%-CI (−0.69, −0.04)] for the goal priming group, *t* (126) = −1.78, *p* = 0.039, *d* = −0.32.

Regarding explicit attitudes toward meat-based nutrition, there were no outliers in the data. There were 64 participants in each group (*N* = 128). Explicit attitudes toward meat-based food were lower in the goal priming intervention group (*M* = 3.35, SD = 1.72) than in the control group (*M* = 3.81, SD = 1.68, see Table 2). However, this difference was not statistically significant [95%-CI (−0.14, 1.05); *t* (126) = 1.52, *p* = 0.066, *d* = 0.27].

### 3.4 Implicit and explicit attitudes as mediators of supermarket food choice

There were no outliers in the data. There were 64 participants in each group (*N* = 128). The amount of chosen meat products during the supermarket task was lower in the goal priming intervention group (*M* = 1.55, SD = 1.89) than in the control group (*M* = 1.94, SD = 1.81, see Table 2). There was no statistically significant difference between chosen meat products of the goal priming group and the control group, with lower meat product scores [95%-CI (−0.26, 1.04)] for the goal priming group, *t* (126) = 1.12, *p* = 0.117, *d* = 0.21.

**Figure 2:**
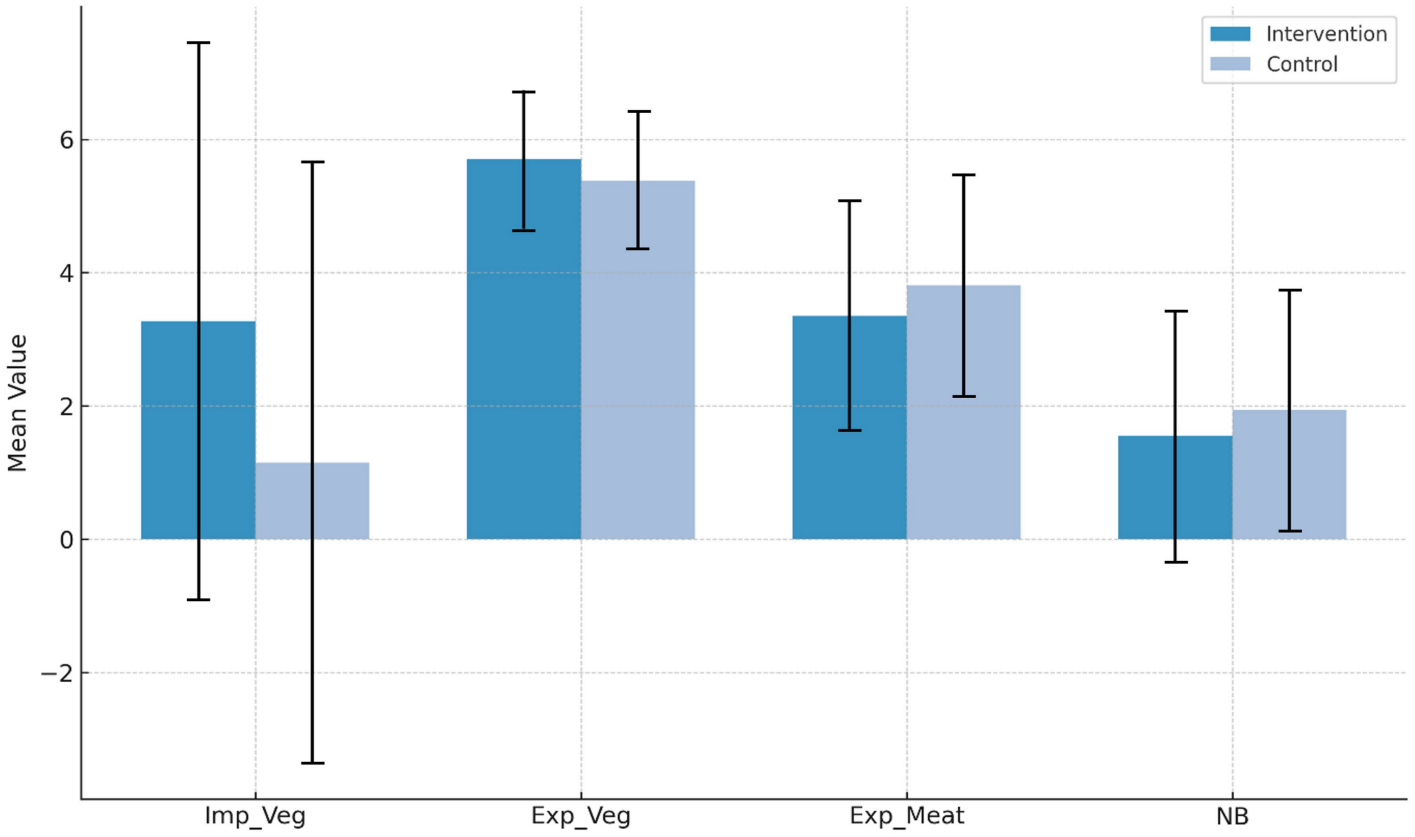
Group comparison of unadjusted means (SD) of implicit attitudes toward vegetarian nutrition (Imp_Veg), explicit attitudes toward vegetarian nutrition (Exp_Veg) and meat-based nutrition (Exp_Meat) and nutritional behavior (NB).

Descriptive plots for the unadjusted means are presented in Figure 2.

Mediation analyses were performed using the PROCESS macro by Hayes (38), which employs ordinary least squares regression to yield unstandardized path coefficients for total, direct, and indirect effects. Bootstrapping with 5,000 samples, together with heteroscedasticity-consistent standard errors (63), was employed to compute the confidence intervals and inferential statistics. Effects were deemed significant when the confidence interval did not include zero.

A simple mediation analysis was performed to investigate whether the goal priming intervention predicts nutritional behavior and whether implicit and explicit attitudes mediate the direct path. A significant effect of goal priming on nutritional behavior was not observed, *B* = −0.047, *p* = 0.770. Because the manipulation did not yield a statistically significant total effect on supermarket choice, indirect effects are reported as process evidence and interpreted with caution, consistent with guidance that prioritizes the indirect effect over the total effect (39, 40).

When implicit attitudes were entered as a mediator, goal priming significantly predicted implicit attitudes, *B* = 2.122, *p* = 0.007, which in turn predicted nutritional behavior significantly, *B* = 0.064, *p* < 0.001. The indirect effect through implicit attitudes was significant, as the 95% confidence interval did not include zero [*ab* = 0.135, 95%-CI (0.029, 0.278)].

When explicit attitudes toward vegetarian food were entered as a mediator, goal priming did not significantly predict explicit attitudes, *B* = 0.325, *p* = 0.078, nor did explicit attitudes significantly predict nutritional behavior, *B* = −0.140, *p* = 0.165. The corresponding indirect effect was also not significant [*ab* = −0.046, 95%-CI (−0.121, 0.022)].

When explicit attitudes toward meat-based food were tested as a mediator, goal priming again did not significantly predict explicit attitudes toward meat-based food, *B* = −0.456, *p* = 0.134. However, explicit attitudes toward meat-based food significantly predicted nutritional behavior, *B* = −0.336, *p* < 0.001. Nevertheless, the indirect effect through explicit attitudes toward meat-based foods was not statistically significant [*ab* = 0.153, 95%-CI (−0.041, 0.387)].

### 3.5 Exploratory analysis

As mentioned, there is a statistically significant difference between the intervention and control groups regarding gender [*χ*^2^(1) = 8.01, *p* = 0.005]. Although gender was not included in the preregistered hypotheses or the original statistical analysis plan (see text footnote 1, respectively), exploratory analyses indicated significant gender differences in implicit attitudes [*t* (123.15) = −5.18, *p* < 0.001], explicit attitudes toward vegetarian nutrition [*t* (126) = −3.41, *p* < 0.001], explicit attitudes toward meat-based nutrition [*t* (126) = 4.37, *p* < 0.001] and nutritional behavior [*χ*^2^ (8) = 25.32, *p* = 0.001], shown in Table 1. To account for the potential confounding role of gender, we added an exploratory extension to each of the main statistical tests by re-running all primary analyses with gender included as a covariate.

Means adjusted for gender showed higher implicit attitudes in the intervention group (*M* = 2.85, SE = 0.51), than in the control group (*M* = 1.57, SE = 0.51, see Table 3) with no statistically significant difference, *F* (1, 125) = 3.00, *p* = 0.086, partial *η*^2^ = 0.023. Regarding explicit attitudes toward vegetarian nutrition, means adjusted for gender showed higher values in the intervention group (*M* = 5.63, SE = 0.13), than in the control group (*M* = 5.45, SE = 0.13, see Table 3), but again no statistically significant difference, *F* (1, 125) = 1.04, *p* = 0.311, partial *η*^2^ = 0.008. Also, means adjusted for gender showed lower explicit attitudes toward meat-based in the intervention group (*M* = 3.50, SE = 0.20), than in the control group (*M* = 3.66, SE = 0.20, see Table 3), still without a statistically significant difference, *F* (1, 125) = 0.29, *p* = 0.591, partial *η*^2^ = 0.002. Regarding nutritional behavior, means adjusted for gender showed nearly the same amount of chosen meat products in the intervention group (*M* = 1.74, SE = 0.22), and in the control group (*M* = 1.74, SE = 0.22, see Table 3) and no statistically significant difference, *F* (1, 125) = 0.00, *p* = 0.999, partial *η*^2^ = 0.000.

**Table 3:** Descriptives of implicit attitudes toward vegetarian nutrition (Imp_Veg), explicit attitudes toward vegetarian (Exp_Veg), meat-based nutrition (Exp_Meat) and nutritional behavior (NB, amount of chosen meat products in supermarket tasks) adjusted for gender.

Variable	Group	*N*	Mean	SE
Imp_Veg	Intervention	64	2.85	0.51
	Control	64	1.57	0.51
Exp_Veg	Intervention	64	5.63	0.13
	Control	64	5.45	0.13
Exp_Meat	Intervention	64	3.50	0.20
	Control	64	3.66	0.20
NB	Intervention	64	1.74	0.22
	Control	64	1.74	0.22

Significant group differences (*p* < 0.05) are indicated with an asterisk (*).

In an extended mediation model, gender was included as a covariate to control for potential confounding effects due to unequal gender distribution across groups.

No significant total effect of goal priming on nutritional behavior was observed, *B* = −0.122, *p* = 0.452, and the direct effect also remained non-significant after accounting for the mediators and the covariate, *B* = −0.166, *p* = 0.228.

When implicit attitudes were included as a mediator, goal priming marginally predicted implicit attitudes, *B* = 1.276, *p* = 0.085. In contrast, implicit attitudes did not significantly predict nutritional behavior, *B* = 0.011, *p* = 0.629. The indirect effect through implicit attitudes in the extended model was not significant, *ab* = 0.014, 95%-CI [−0.048, 0.076].

For explicit attitudes toward vegetarian food, goal priming did not significantly predict the mediator, *B* = 0.186, *p* = 0.321, nor did the mediator significantly predict nutritional behavior, *B* = −0.134, *p* = 0.238. The indirect effect via this path was also not significant, *ab* = −0.025, 95%-CI [−0.095, 0.032].

Explicit attitudes toward meat-based food were not significantly predicted by goal priming, *B* = −0.158, *p* = 0.592, but still significantly predicted nutritional behavior, *B* = −0.345, *p* < 0.001. Still, the indirect effect via this path did not reach significance, *ab* = 0.054, 95%-CI [−0.138, 0.269].

## 4 Discussion

This study aimed to investigate changes in explicit and implicit attitudes toward vegetarian and meat-based nutrition through goal priming focused on vegetarian nutrition and its impact on actual nutritional behavior.

### 4.1 Goal priming and implicit attitudes toward vegetarian nutrition

The results of this study indicate that goal priming has a significant influence on implicit attitudes toward vegetarian nutrition. Participants in the intervention group demonstrated more positive implicit attitudes than those in the control group, supporting Hypothesis 1. This aligns with the concept of *evaluative readiness,* which refers to the temporary shift in automatic valuations following goal priming. When a goal becomes active, either consciously or non-consciously, positive associations with goal-relevant stimuli become more accessible, resulting in more favorable affective responses (17, 18). The ability to alter implicit attitudes through a brief priming intervention supports assumptions from dual-process theories. According to ART (7), type-1 processes are fast, affective, and associative, serving as the initial input for behavior. In this framework, implicit attitudes are considered the outcome of automatic affective valuations that occur before reflective evaluations (type-2 processes). The present study demonstrates that these type-1 processes can indeed be modulated by activating a long-term goal, in this case, sustainable nutrition, through a subtle linguistic priming task.

The change in implicit attitudes, although not directly linked to behavioral outcomes in the present study, holds substantial theoretical and practical relevance. First, more positive implicit valuations enhance the accessibility of favorable automatic associations with vegetarian food, which can increase the likelihood of vegetarian choices in fast or habitual decision contexts (18). Second, implicit attitudes are known to influence behavior, particularly when cognitive resources are limited, such as under time pressure, distraction, or ego depletion (41). Even if not behaviorally evident here, such changes may act as a latent mechanism for future decisions.

Moreover, a shift in implicit attitudes may reduce cognitive dissonance between reflective values (e.g., sustainability goals) and affective tendencies, potentially increasing openness to future behavior change. In the long run, repeated activation of positive implicit automatic associations could also influence explicit attitudes, as type-1 and type-2 processes are interlinked over time (7, 42). Affective changes may thus provide an emotional foundation for the development of more consistent and internalized pro-vegetarian beliefs.

### 4.2 Goal priming and explicit attitudes toward vegetarian and meat-based nutrition

In line with Hypothesis 2a, results revealed a small but statistically significant increase in explicit attitudes toward vegetarian food in the goal priming group compared to the control group. However, explicit attitudes toward meat-based food did not differ significantly between groups (H2b). These findings suggest a partial support for Hypothesis 2, indicating that goal priming has influenced reflective evaluations of vegetarian options, but not those of meat-based ones.

The observed shift in explicit attitudes toward vegetarian nutrition carries meaningful implications, particularly within the framework of the ART (7). As explicit attitudes result from type-2 processes—conscious, propositional reasoning—they are based on beliefs, norms, and internalized values (21). Therefore, even subtle semantic priming may reinforce pre-existing sustainability-related beliefs or prompt individuals to reassess the benefits of vegetarian eating.

Positive shifts in explicit evaluations can play an essential role in long-term behavior regulation. Unlike affective valuations, which may dominate in spontaneous decisions, explicit attitudes are more predictive of behavior under conditions that allow for deliberation and self-reflection (8, 42). Thus, even modest increases in explicit attitudes of vegetarian nutrition could strengthen behavioral intentions and contribute to more consistent dietary choices or sustainable nutrition over time, especially when coupled with planning and self-regulatory resources (43).

In contrast, the absence of a significant effect on explicit attitudes toward meat-based food raises questions. One possible explanation is that explicit attitudes toward meat are more entrenched, often linked to identity or cultural traditions (44). Research suggests that meat consumption is sometimes justified through cognitive strategies such as the “4Ns” (necessary, natural, normal, nice), which can buffer against attitudinal change (45). Although the intervention included negative information about meat production, such input may have evoked reactance or dissonance, leading to defensive processing rather than attitude change (46). Moreover, participants may have had stronger motivational reasons to defend existing positive views of meat-based meals, especially if meat consumption is perceived as normative in their social environment (64), as it may be the case in our sample consisting only of sport students, as meat is one of the most important protein sources, with high biological value that can satisfy metabolic muscular necessities of sport practitioners (65).

While priming may be suitable for enhancing openness to vegetarian options, its capacity to reduce support for socially and emotionally significant behaviors like meat-eating may be limited, particularly in one-shot interventions.

### 4.3 The moderating role of eating habits in implicit and explicit attitudes toward vegetarian and meat-based nutrition

Contrary to Hypothesis 3a, the goal priming intervention did not result in significantly different nutritional behavior between the experimental and control group. While participants in the priming group selected fewer meat products on average, this difference did not reach statistical significance. Thus, the intervention failed to elicit a measurable behavioral change in the immediate context of the online supermarket task.

However, mediation analyses yielded partially supportive evidence for Hypothesis 3b. Specifically, goal priming significantly increased implicit attitudes toward vegetarian nutrition, and these in turn predicted lower meat product selection. The indirect effect via implicit attitudes was statistically significant, suggesting that the intervention influenced behavior through a subtle, affect-driven mechanism. In contrast, neither explicit attitudes toward vegetarian nor meat-based food significantly mediated the relationship between priming and behavior.

Interestingly, while the goal priming intervention did not significantly affect explicit attitudes toward meat, these attitudes consistently predicted participants’ food choices in the supermarket task. Across multiple models, more negative explicit evaluations of meat were associated with lower selection of meat products, independent of group assignment. This finding highlights the enduring role of propositional evaluations.

Although the supermarket paradigm simulates a real-world decision context, participants completed it in a digital, low-stakes environment with no real consequences or social pressures. Prior research has shown that implicit processes are more likely to influence behavior under conditions of cognitive load, time pressure, or distraction. It is possible that the task was too controlled and reflective, favoring deliberation over spontaneous choice.

The findings also raise questions about the strength and persistence of the priming manipulation. While it was sufficient to shift implicit attitudes, it may not have been intense or durable enough to alter behavior. Nonetheless, the significant indirect effect via implicit attitudes suggests that priming interventions can initiate affective precursors of behavior, which may unfold over time or under more automatic conditions.

### 4.4 The role of gender

Although gender was not included in the preregistered hypotheses or the original statistical analysis plan, exploratory analyses revealed significant gender imbalances between conditions and consistent gender differences across key outcome variables. The gender related findings should be interpreted with caution given the single session design, the achieved sample size, and the absence of pre-intervention baselines. As noted, brief gender adjusted robustness checks did not change the direction of the preregistered effects. Specifically, women scored significantly higher than men in implicit and explicit attitudes toward vegetarian food, lower in explicit attitudes toward meat, and chose fewer meat products overall. These differences are consistent with a growing body of literature indicating gender differences in pro environmental dietary patterns. Across countries, men report higher meat intake whereas women show greater openness to vegetarian eating and other pro environmental nutrition practices, with effects partly shaped by gender role beliefs and identity processes (47). Large scale and cross-national studies further show that gender gaps in meat consumption and sustainable diet intentions vary by context and can widen in more gender equal and highly developed settings, underscoring cultural moderation of these patterns (48). Evidence from European samples also documents that women more often adopt pro environmental nutrition practices such as eating vegetarian and opting for organic products (49) and that vegetarian identities and motivations differ by gender (50).When gender was statistically controlled as a covariate in all primary analyses, nearly all effects of the goal priming intervention were attenuated, including the significant differences in implicit and explicit attitudes. This raises the question of whether gender is a more powerful predictor of sustainability-related behavior than the priming intervention itself. While the intervention had small-to-moderate effects on implicit and explicit attitudes, the effect sizes for gender-related differences were larger, more consistent and extended to actual food choice behavior. This observation points to important implications for intervention design and public health messaging. Suppose men show significantly less favorable implicit and explicit attitudes toward plant-based eating and are less likely to choose vegetarian options. In that case, it may be essential to target interventions that specifically improve implicit attitudes among men.

However, gender is unlikely to be the only relevant moderator. Other individual differences, such as cultural background, food-related identity (vegetarian, flexitarian, omnivore), moral values, political ideology, or even health orientation may likewise influence how people respond to goal priming or sustainability messaging. The current study focused on gender due to its empirical salience in the data and the clear imbalance across conditions, but future work should explore broader interaction models that include multiple sociocultural and psychological moderators (e.g., value-based motivation, dietary habits, environmental concern).

### 4.5 Limitations and future research

To the best of our knowledge, this is the first study to investigate the effect of short-term goal priming on implicit and explicit attitudes toward sustainable nutrition in the context of ART (7). The RCT design offers several key advantages that strengthen the validity and reliability of the results.

Several limitations of the present study warrant careful consideration. First, our vegetarian category in the explicit ratings and the IAT included a small mixed set with two dairy based items and three strictly plant-based items. While this reflects a common usage of the term vegetarian, dairy items can differ from plant-based alternatives in life cycle environmental impacts and nutritional profiles and may elicit distinct consumer perceptions. Given the small number of images, we could not stratify analyses by dairy versus plant-based subgroups. Future studies should balance these subgroups or use strictly plant-based stimuli to improve interpretability. Furthermore, the study employed a single-session priming intervention with immediate post-measurement of attitudes and behavior. While goal priming has been shown to alter automatic valuations in the short term (18), it remains unclear how durable and behaviorally impactful such changes are without reinforcement. Future research should examine long-term effects, for instance, through follow-up assessments or repeated priming sessions, to better understand the persistence of implicit and explicit attitude shifts. Additionally, the study lacked a pre-post design, which made it challenging to detect intraindividual change and assess baseline equivalence between groups. A repeated-measures design would enable more robust causal inference and clarify whether observed differences truly reflect change due to the intervention rather than pre-existing group differences.

Furthermore, several methodological and contextual factors may have weakened the effects of goal priming. For one, the explicit attitudes toward vegetarian food showed relatively high baseline scores, suggesting a possible ceiling effect that limited the potential for further positive change. In contrast, explicit attitudes toward meat may be more culturally embedded, requiring stronger or more emotionally engaging interventions to be meaningfully shifted. A brief, text-based priming may have been too subtle or too short to challenge well-established propositional evaluations about meat, especially in participants with habitual meat consumption patterns.

Moreover, the study relied on a delayed, low-reactivity comprehension and manipulation check rather than an immediate measure of goal accessibility. This approach reduces the likelihood of hypothesis awareness but does not provide a direct index of goal activation.

The IAT target labels denoted food types (“vegetarian,” “meat”), yet some readers may still construe vegetarian as a dietary identity; future work should display fully parallel basic-level labels (e.g., “vegetarian food” vs. “meat-based food”) on screen to minimize any residual ambiguity.

Explicit ratings were always collected before the implicit measure. A fixed order can reduce the sensitivity of the IAT and may introduce carryover or habituation effects from earlier tasks.

Also, item level ratings for the explicit composites were not preserved in the final analysis file, which precludes reporting internal consistency for these indices. Future work should retain item level data to permit reliability estimation alongside composite scores.

The behavioral outcome was assessed with a simulated supermarket task that captured immediate category and product choices within a single laboratory session. The task did not involve real purchases or consumption and was not incentive compatible, and no pre-intervention baseline or follow-up assessment was available. Although random assignment mitigates pre-existing differences, these features limit ecological validity and constrain inferences about sustained behavior change. Consequently, behavioral interpretations should be regarded as provisional and specific to the simulated setting.

Besides, analyses were conducted in the full sample rather than restricting to omnivores. This choice preserves external validity and statistical power and is consistent with the goal-priming manipulation, which targets sustainable eating goals irrespective of prior diet. Nevertheless, pre-existing dietary habit may moderate responses. Future studies should consider designs that balance dietary groups *a priori* or restrict to omnivores and preregister subgroup and moderation tests by eating habit to reduce interpretational ambiguity. Unequal group sizes for omnivores versus vegetarian or vegan participants limit the interpretability of direct comparisons by eating habit. Future research should recruit balanced strata by eating habit or restrict analyses to omnivores when diet is a focal moderator. Although instructions and tasks were standardized, the mere presence or perceived expectations of the researcher may have subtly influenced participants’ responses, particularly regarding socially desirable topics such as sustainability. Future research should consider blind administration or digital automation of the entire procedure to minimize such effects.

Additionally, the sample consisted solely of students of applied movement science, a population that may differ from the general public in terms of health awareness, nutritional knowledge, and educational background.

Momentary hunger was not recorded. Hunger can influence responsiveness to food cues and may add unexplained variance to both implicit and explicit evaluations as well as to food choice (51). Although random assignment should mitigate systematic bias between conditions, future studies should include a brief hunger rating, consider standardizing pre-session instructions, and, where feasible, adjust analyses for hunger and related contextual factors such as time of day.

Lastly, gender was the only moderator that was exploratively analyzed in this study, primarily due to the observed imbalance across the groups. However, it is likely not the sole meaningful moderator of intervention effects. Other variables, such as dietary identity (e.g., flexitarian, omnivore), environmental concern, moral values, or political ideology, may also influence receptiveness to priming. Future work should employ multivariate moderation and mediation models to more effectively capture the interplay between individual characteristics and reflective-affective intervention pathways. In the present study, the analysis strategy was restricted to the preregistered independent samples t-tests across tasks. Although multivariable or mixed effects models can integrate moderators and interactions, such models were not preregistered and the available sample size afforded limited power for interaction terms. The inclusion of gender as a covariate therefore constitutes an exploratory deviation motivated by pronounced group differences. Future studies should preregister integrated models that include gender and eating habit and recruit sufficiently large samples to test interactions with adequate power.

The pattern of improved implicit and explicit attitudes after environmental goal priming, together with tentative indirect paths to choice and the absence of a total effect on the supermarket outcome, suggests cautious and targeted applications. Very brief goal cues that pair one salient environmental fact with one actionable tip could be tested at the point of choice in online supermarket interfaces or on shelf labels to raise the salience of plant-based options without increasing cognitive load. Given the exploratory gender differences, wording should be neutral and broadly appealing, with optional deeper information for interested users. Field studies with real outcomes are needed to evaluate durability and practical impact.

## 5 Conclusion

This study examined the impact of goal priming on implicit and explicit attitudes toward vegetarian and meat-based diets, as well as subsequent food choice behavior. Grounded in dual-process models such as the Affective-Reflective Theory (7) and the concept of evaluative readiness (17), the results provide differentiated support for the proposed hypotheses.

The findings demonstrate that even brief goal priming can effectively increase implicit attitudes toward vegetarian food, suggesting that automatic affective processes are malleable and responsive to contextual cues. Moreover, a small but significant improvement in explicit attitudes toward vegetarian food was observed, highlighting the potential of goal priming to strengthen reflective, propositional evaluations. However, explicit attitudes toward meat-based food remained unaffected, and no direct behavioral effects were found in the simulated shopping task.

Nevertheless, the mediation analysis revealed that goal priming indirectly influenced behavior through changes in implicit attitudes, underlining the functional relevance of automatic processes in sustainable decision-making. Additionally, explicit attitudes toward meat consistently predicted food choices, emphasizing the role of both affective and reflective systems in guiding behavior, which supports the necessity of dual-process models. Exploratory analyses further revealed that gender emerged as a significant determinant of both attitudes and behavior, potentially outweighing the influence of the intervention itself.

The results provide theoretical support for dual-process accounts of behavior change and offer practical ways to promote sustainable food choices through subtle and short-term goal priming.

## Data Availability

The datasets presented in this study can be found in online repositories. The names of the repository/repositories and accession number(s) can be found at: Data and material are stored at OSF: https://osf.io/hmkcn.

## Footnotes

^1^ https://osf.io/hmkcn/
