# Supplementary material for: Affective and reflective attitudes toward vegetarian food consumption: the effect of goal priming
Source: Front Nutr. 2025 Nov 6;12:1653935. doi: 10.3389/fnut.2025.1653935 (PMC12631265; doi:10.3389/fnut.2025.1653935)
Supplement: Supplementary file 1 [file Table_1.docx]

**Supplementary Table 1**

Image Number of Blechert et al. (2019), description, category, energy density in kilocalories per 100 g (Kcal 100g), the database complexity index (complexity), and overall means of familiarity, valence and arousal of the used stimuli

| **Image** | **Description** | **Category** | **Kcal 100g** | **Complexity** | **Familiarity** | **Valence** | **Arousal** |
| --- | --- | --- | --- | --- | --- | --- | --- |
| 301 | roasted chicken | meat | 140 | 0.48 | 86.98 | 38.21 | 24.15 |
| 485 | burger patty with french fries and salad | meat | 199.6 | 0.45 | 99.54 | 38.86 | 23.64 |
| 552 | roast beef | meat | 120 | 0.19 | 98.04 | 51.18 | 33.64 |
| 563 | steak | meat | 107.5 | 0.40 | 94.39 | 48.69 | 35.28 |
| 721 | steak | meat | 121 | 0.25 | 98.44 | 55.50 | 52.95 |
| 325 | fruit salad | vegetarian | 53 | 0.35 | 100 | 81.60 | 56.90 |
| 526 | plate of salad | vegetarian | 47.3 | 0.42 | 99.83 | 67.03 | 44.03 |
| 567 | tomato and mozzarella | vegetarian; dairy present | 86.7 | 0.29 | 92.80 | 67.74 | 43.07 |
| 705 | pasta with white sauce | vegetarian; dairy present | 170 | 0.41 | 100 | 66.65 | 45.75 |
| 715 | panini | vegetarian | 212 | 0.42 | 98.28 | 71.73 | 31.43 |
